# *Arachis hypogaea* L. stem and leaf extract improves the sleep behavior of pentobarbital-treated rats

**DOI:** 10.3892/br.2014.259

**Published:** 2014-03-19

**Authors:** XIAO-YAN ZU, GUANG-QUAN XIONG, SHENG-RONG GENG, TAO LIAO, XIN LI, ZHEN-YA ZHANG

**Affiliations:** 1 Institute for Farm Products Processing and Nuclear-Agricultural Technology, Hubei Academy of Agricultural Sciences, Wuhan, Hubei 430064, P.R. China; 2 Graduate School of Life and Environmental Sciences, University of Tsukuba, Tsukuba, Ibaraki 305-8572, Japan

**Keywords:** *Arachis hypogaea* L. stem and leaf extract, sleep behaviors, pentobarbital-treated rats

## Abstract

This study was conducted to evaluate the sedative effects of *Arachis hypogaea* L. stem and leaf extract (AHSLE) and determine its effect pathways through γ-aminobutyric acid (GABA)-gated channels on male Sprague-Dawley rats treated with pentobarbital. AHSLE was obtained from 98°C water (3 h, extracted twice). AHSLE and flumazenil (a GABA type A receptor antagonist) were administered to the rats orally, whereas pentobarbital sodium and muscimol (a GABA type A receptor agonist) were administered intraperitoneally (i.p.). The results demonstrated that AHSLE decreased sleep latency and increased sleep time in pentobarbital-treated rats (50 mg/kg, i.p.). The coadministration of AHSLE and muscimol (0.05 mg/kg) significantly increased sleep time and reduced sleep latency in pentobarbital-treated rats and these actions were significantly antagonized by flumazenil at a dose of 3.5 mg/kg. These results indicated that AHSLE improved the sleep behavior in pentobarbital-treated rats, possibly through GABA-gated channel-related mechanisms.

## Introduction

*Arachis hypogaea* L. stem and leaf extract (AHSLE) is a type of sleep aid used in China (1–3). γ-aminobutyric acid (GABA) receptors are known to play an important role in the modulation of barbiturate-induced sleep through interaction with GABAergic systems (4,5). We previously reported (6) that AHSLE may lead to a significant decrease of glutamate/GABA ratio in corresponding brain areas in freely behaving rats. These findings raised the questions of whether AHSLE results in external manifestations of rat sleep behavior and whether AHSLE modulates barbiturate-induced sleep through GABAergic channels.

In this study, in order to evaluate the sedative effects of AHSLE on rats and investigate its possible mechanisms of action, male Sprague-Dawley (SD) rats were employed and sleep was induced by pentobarbital. Sleep time and sleep latency were recorded following AHSLE administration. Muscimol, a GABA type A (GABA_A_) receptor agonist, and flumazenil, a GABA_A_ receptor antagonist, were used as positive controls, respectively.

## Materials and methods

### Plant, AHSLE and reagents

The *Arachis hypogaea* L. plants were collected from the shores of lake Yezi (Wuhan, Hubei, China) in August, 2011. The plant was authenticated by the Agriculture and Forestry Research Center of Tsukuba University, Japan. AHSLE powder was obtained as previously described (6,7), comprising 1.92% protein and 65.31% carbohydrate. All the reagents used were of the highest available purity. Pentobarbital sodium was purchased from Rejuvenation Pharmaceutical Co., Ltd., (Fuzhou, China). Muscimol was purchased from Sigma (St. Louis, MO, USA). Flumazenil and other chemicals used were purchased from Wako Pure Chemical Industries, Ltd., (Osaka, Japan).

### Animals

Male SD rats, aged 8 weeks and weighing 270±30 g, were provided by the Laboratory Animal Resource Center, University of Tsukuba, Japan. The animals were housed at ambient conditions of 25°C, with 12-h light/dark cycles (light on at 08:00, light off at 20:00) and were given *ad libitum* access to food and water. All the animal experiments were conducted humanely, following approval from the Institutional Animal Experiment Committee of Tsukuba University, Japan and in accordance with the regulations for Animal Experiments and Fundamental Guidelines under the jurisdiction of the Japanese Ministries of Education, Culture, Sports, Science and Technology.

### Experimental protocols

The experiments were performed between 20:00 and 24:00. Rats (n=48) were employed and habituated in an animal lab for at least 7 days, then randomly divided into groups. In the 4 experiments, the number of rats in each group was 12, 9–12, 12 and 8, respectively. All the rats were reused after 2 weeks. The animals were fasted for 24 h prior to the experiment. AHSLE in physiological saline and flumazenil in 10% dimethyl sulfoxide were administered orally to the rats, whereas muscimol was administered intraperitoneally (i.p.). At 30 min following the administration of muscimol, flumazenil or AHSLE, pentobarbital sodium in physiological saline was administered i.p. to each rat to induce sleep. The animals that remained immobile for >1 min were considered to be asleep. The sleep latency was recorded from the pentobarbital injection to 1 min after the loss of the righting reflex and the sleep time was recorded from 1 min after the loss of the righting reflex to recovery (8).

### Statistical analysis

The obtained data were analyzed using two-tailed Student’s t-test (version 2003, Microsoft excel) for comparisons and the results are expressed as means ± SD. P<0.05 was considered to indicate statistically significant differences.

## Results

### Sedative effects of pentobarbital, AHSLE and muscimol

The results (Table I) revealed that pentobarbital induced sleep at a dosage of 50 mg/kg. Muscimol was unsuccessful in inducing sleep. AHSLE alone, similar to muscimol, was unable to induce sleep directly, even at a high dose (500 mg/kg).

### Effects of AHSLE on sleep in pentobarbital-treated rats

**Figure 1:**
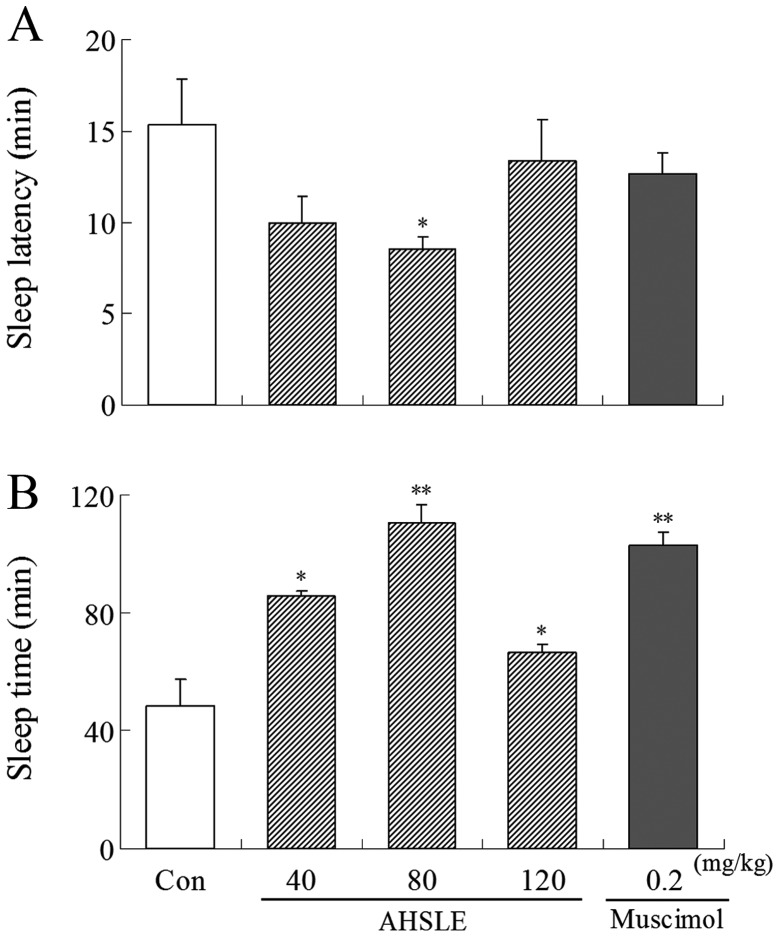
Effects of *Arachis hypogaea* L. stem and leaf extract (AHSLE) on sleep in pentobarbital-treated rats. Following administration of muscimol or AHSLE, pentobarbital (50 mg/kg) was intraperitoneally administered to the rats. (A) Sleep latency and (B) sleep time were recorded. ^*^P<0.05 and ^**^P<0.01, compared to the control (Con, open bar, without muscimol and AHSLE).

Following administration of muscimol or AHSLE, pentobarbital (50 mg/kg) was administered i.p. to the rats. AHSLE decreased sleep latency and prolonged sleep time in rats, particularly at a dose of 80 mg/kg, following sleep induction by pentobarbital. Muscimol (0.2 mg/kg) as a positive control also induced a decrease in sleep latency and a prolongation of the total sleep time (Fig. 1).

### Effects of AHSLE with muscimol on sleep in pentobarbital-treated rats

**Figure 2:**
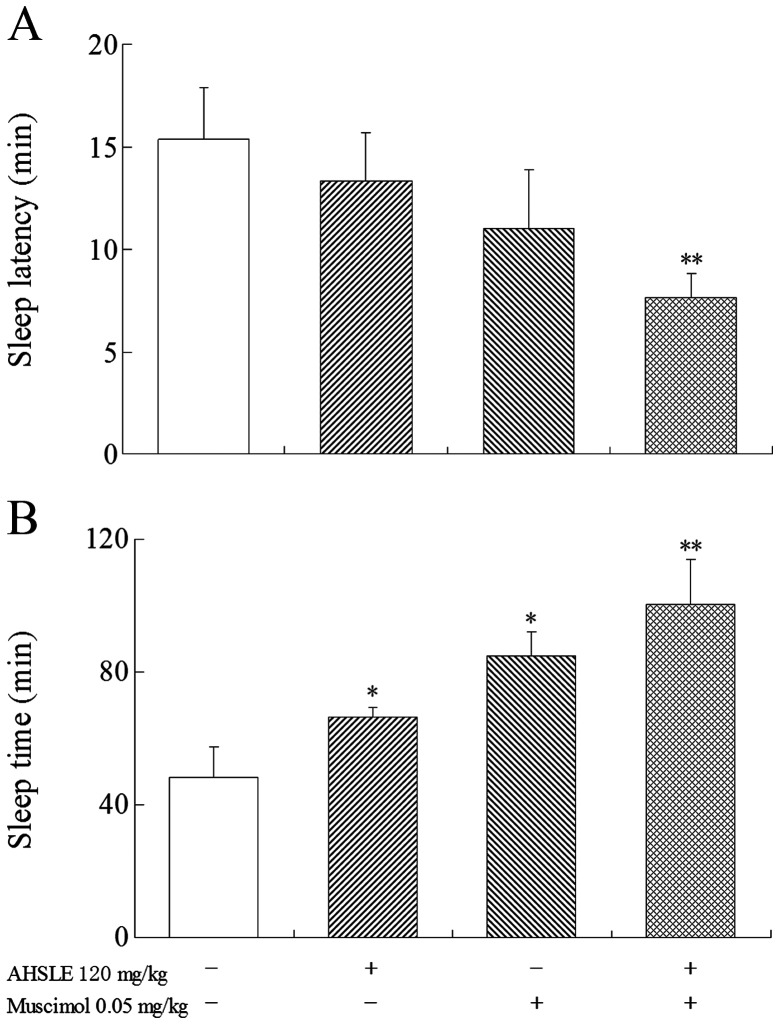
Effects of *Arachis hypogaea* L. stem and leaf extract (AHSLE) and muscimol on sleep in pentobarbital-treated rats. Following administration of AHSLE (120 mg/kg) and muscimol (0.05 mg/kg), pentobarbital (50 mg/kg) was intraperitoneally administered to the rats. (A) Sleep latency and (B) sleep time were recorded. Each column represents the mean ± SD. ^*^P<0.05 and ^**^P<0.01, compared to the control (Con, open bar, without muscimol and AHSLE).

Prior to the administration of pentobarbital (50 mg/kg), low doses of muscimol (0.05 mg/kg) were used in the AHSLE (120 mg/kg)-treated groups (Fig. 2) to observe the direct association between the effects of muscimol and those of AHSLE. The results demonstrated that the pentobarbital-treated rats in the AHSLE+muscimol group exhibited significantly (P<0.01) increased sleep time and reduced sleep latency.

### Effects of AHSLE with flumazenil on sleep in pentobarbital-treated rats

**Figure 3:**
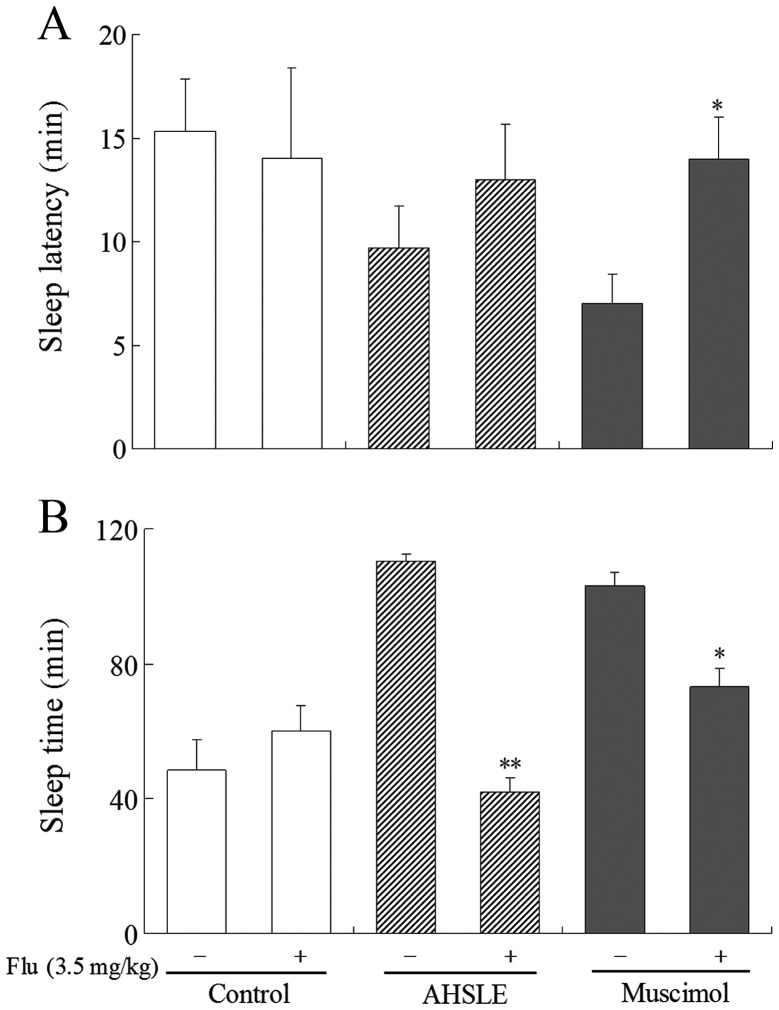
Effects of *Arachis hypogaea* L. stem and leaf extract (AHSLE) and flumazenil on sleep in pentobarbital-treated rats. Following administration of AHSLE (80 mg/kg), muscimol (0.2 mg/kg) or flumazenil (Flu, 3.5 mg/kg), pentobarbital (50 mg/kg) was intraperitoneally administered to the rats. (A) Sleep latency and (B) sleep time were recorded. Data are presented as means ± SD (n=12). ^*^P<0.05 and ^**^P<0.01, compared to the group without Flu.

In pentobarbital (50 mg/kg)-treated rats, AHSLE decreased sleep latency and also significantly prolonged sleep time at doses of 80 mg/kg (Fig. 1A and B). These actions were significantly antagonized by flumazenil, a benzodiazepine receptor antagonist, at a dose of 3.5 mg/kg (Fig. 3A and B). This antagonistic effect of flumazenil was also observed in rats that were administered muscimol.

## Discussion

GABA is a type of inhibitory neurotransmitter amino acid in the brain, is primarily formed from glutamate via the action of glutamate decarboxylase (9) and may be used as a parameter to characterize the sleep-wake cycle (10). Pentobarbital is known to potentiate the effects of GABA through acting on the receptor sites of the GABA/benzodiazepine receptor-ionophore complex (11). The sedative effects may be assessed by decreases in the pentobarbital-induced sleep time and are possibly mediated through GABAergic systems (12). In this study, we aimed to determine whether sleep enhancement following AHSLE administration is mediated via the GABAergic systems.

The effects of different doses of AHSLE on rats with or without pentobarbital treatment were investigated. We observed that AHSLE was able to prolong pentobarbital-induced sleep at a lower dose (40 mg/kg); however, it was unable to induce sleep alone, even at a significantly higher dosage (500 mg/kg), which was similar to the effects of the GABA_A_ receptor agonist muscimol. Moreover, AHSLE acted synergistically with muscimol in pentobarbital-treated rats. It was indicated that GABA_A_ receptor channels may be involved in the AHSLE sedative effect pathways.

Previous studies demonstrated that flumazenil decreases sleep time and increases waking and sleep latency through suppressing the effects of GABA_A_ receptors (13,14). To further investigate the mechanisms underlying the potentiation of sedation caused by AHSLE, the effects of flumazenil on sleep induced by AHSLE in pentobarbital-treated rats were investigated. Flumazenil exerted no obvious effect when used alone, whereas it exhibited a significant antagonistic effect to that of AHSLE in pentobarbital-treated rats. These results demonstrated that the synergistic effects of AHSLE with pentobarbital or muscimol may antagonize the effects of flumazenil, a GABA_A_ receptor antagonist. These findings also suggested that the activation of GABAergic systems induced by AHSLE may potentiate the activity of sedative agents.

We analyzed the essential oil components of AHSLE according to previous studies (15,16) and found that it contained linalool (7), which may contribute to the sedative effects of AHSLE. The role of linalool requires validation by further experiments.

In conclusion, our results indicated that AHSLE enhanced the sedative effects in pentobarbital-treated rats and these effects were possibly mediated through GABA-gated channels and benzodiazepine receptors. AHSLE may be a candidate agent for the management of sleep disorders. However, the pharmacological effects of AHSLE derivatives, such as linalool, require further investigations.

## Figures and Tables

**Table I:** Effects of pentobarbital, AHLAE and muscimol on rats.

Groups	Dose (mg/kg)	No. falling asleep/total	Sleep latency (min)	Sleep time (min)
Pentobarbital	30	5/12	31.67±1.15	41.33±1.53
Pentobarbital	50	12/12	15.33±2.52	48.33±9.07
AHSLE	500	0/12	0	0
Muscimol	10	0/12	0	0

The rats were fasted for 24 h prior to the experiment. AHSLE, muscimol and pentobarbital were administered and the number of rats falling asleep/total number, sleep latency and sleep time were recorded. The data of sleep latency and sleep time are presented as means ± SD. AHSLE, *Arachis hypogaea* L. stem and leaf extract.
